# Continuous monitoring of vital sign abnormalities; association to clinical complications in 500 postoperative patients

**DOI:** 10.1111/aas.14048

**Published:** 2022-02-28

**Authors:** Camilla Haahr‐Raunkjaer, Jesper Mølgaard, Mikkel Elvekjaer, Søren M. Rasmussen, Michael P. Achiam, Lars N. Jorgensen, Mette I.V. Søgaard, Katja K. Grønbæk, Anne‐Britt Oxbøll, Helge B. D. Sørensen, Christian S. Meyhoff, Eske K. Aasvang

**Affiliations:** ^1^ Department of Anaesthesiology Centre for Cancer and Organ Diseases Rigshospitalet University of Copenhagen Copenhagen Denmark; ^2^ Department of Anaesthesia and Intensive Care, Bispebjerg and Frederiksberg Hospital University of Copenhagen Copenhagen Denmark; ^3^ Copenhagen Centre for Translational Research Copenhagen University Hospital Bispebjerg and Frederiksberg Copenhagen Denmark; ^4^ Biomedical Engineering Department of Health Technology Technical University of Denmark Lyngby Denmark; ^5^ Department of Surgical Gastroenterology Centre for Cancer and Organ Diseases Rigshospitalet University of Copenhagen Copenhagen Denmark; ^6^ Digestive Disease Centre, Bispebjerg and Frederiksberg Hospital University of Copenhagen Copenhagen Denmark; ^7^ Department of Clinical Medicine University of Copenhagen Copenhagen Denmark

**Keywords:** abnormal vital signs, continuous monitoring, early warning score, postoperative complications, serious adverse events, wearable, wireless devices

## Abstract

**Background:**

Patients undergoing major surgery are at risk of complications, so‐called serious adverse events (SAE). Continuous monitoring may detect deteriorating patients by recording abnormal vital signs. We aimed to assess the association between abnormal vital signs inspired by Early Warning Score thresholds and subsequent SAEs in patients undergoing major abdominal surgery.

**Methods:**

Prospective observational cohort study continuously monitoring heart rate, respiratory rate, peripheral oxygen saturation, and blood pressure for up to 96 h in 500 postoperative patients admitted to the general ward. Exposure variables were vital sign abnormalities, primary outcome was any serious adverse event occurring within 30 postoperative days. The primary analysis investigated the association between exposure variables per 24 h and subsequent serious adverse events.

**Results:**

Serious adverse events occurred in 37% of patients, with 38% occurring during monitoring. Among patients with SAE during monitoring, the median duration of vital sign abnormalities was 272 min (IQR 110–447), compared to 259 min (IQR 153–394) in patients with SAE after monitoring and 261 min (IQR 132–468) in the patients without any SAE (*p *= .62 for all three group comparisons). Episodes of heart rate ≥110 bpm occurred in 16%, 7.1%, and 3.9% of patients in the time before SAE during monitoring, after monitoring, and without SAE, respectively (*p *< .002). Patients with SAE after monitoring experienced more episodes of hypotension ≤90 mm Hg/24 h (*p *= .001).

**Conclusion:**

Overall duration of vital sign abnormalities at current thresholds were not significantly associated with subsequent serious adverse events, but more patients with tachycardia and hypotension had subsequent serious adverse events.

**Trial registration:**

Clinicaltrials.gov, identifier NCT03491137.

**Editorial Comment**

To try to improve perioperative safety. One possible means could be continuous monitoring of vital signs in the early postoperative period and outside of the operating room and post‐anesthesia areas. This study found that overall duration of abnormalities was not associated with serious adverse events, when vital sign thresholds inspired by early warning scores.

## 1 INTRODUCTION

Despite advancements in postoperative outcomes over the last decades due to optimized anesthesia and surgical techniques,^1^ up to 20%–30% of patients undergoing major abdominal surgery are at risk for developing postoperative serious adverse events (SAEs) within 30 days, such as surgical site infection, pneumonia, and myocardial infarction.^2^, ^3^, ^4^ Furthermore, with a rapidly increasing number of surgical procedures (globally 230 million in 2004 and 313 million in 2012)^5^, ^6^ and the fact that if postoperative death were considered a disease, it would be the world's third leading contributor of death,^7^ relates SAEs to have significant negative implications not only for patients but also economically.^8^

Patients are monitored to reduce the frequency of complications based on the assumption that physiologic vital sign abnormalities such as tachycardia, hypotension, and desaturation precede adverse events before becoming clinically evident.^9^, ^10^ Failure to rescue may be due to the lack of recognition and subsequent delay in interventions.^11^ Therefore, so‐called track and trigger systems were implemented across European countries and the United States to track abnormal vital signs and trigger a treatment escalation protocol for deteriorating patients. As example is the Early Warning Score (EWS) systems,^12^, ^13^ consisting of manual monitoring at intervals up to 8 h, potentially leaving the patient unobserved for most of the day, where complications may occur between the measurements. This fundamental shortcoming may explain the lack of impact on morbidity and mortality from EWS and similar manual systems.^14^, ^15^ Consequently, future monitoring of patients at the general ward will need to replace the EWS and matching systems, potentially by automated continuous wireless monitoring.

Continuous wireless monitoring may enable high‐frequency monitoring of physiological status on general wards outside the intensive care (ICU) or post‐anesthesia care unit (PACU) without restricting early postoperative mobilization or requesting unrealistic staffing. Ongoing technological research on general wards has consistently shown continuous monitoring to be superior to track and trigger systems in detecting abnormal vital signs.^16^, ^17^, ^18^

However, before such technology is implemented, it is imperative to identify which physiologic vital sign thresholds or patterns are associated with severe clinical outcomes as the alerting technology otherwise would pose unnecessary work to staff due to recording self‐limiting or unimportant vital sign abnormalities. Therefore, this study assessed abnormal vital signs inspired by the routinely used EWS thresholds and applied them to continuous monitoring.

We investigated the duration and frequency of postoperative vital sign abnormalities detected by a wireless body sensor network in patients undergoing major abdominal surgery. The aim was to explore if severe abnormal vital signs were associated with the subsequent development of SAE. We hypothesized that vital sign abnormalities occurred more prolonged and more often in patients with SAE. Four standard vital signs, heart rate (HR), respiratory rate (RR), blood pressure (BP), and peripheral oxygen saturation (SpO_2_), constituted the exposure variables, the primary outcome being any SAE occurring within 30 postoperative days.

## 2 METHODS

This prospective observational cohort study was registered at http://ClinicalTrials.gov (NCT03491137) after written approval from the Regional Ethics Committee, Copenhagen, Denmark, February 2018 (H‐17033535). All patients gave written informed consent before participating in the study. The study is a part of the Wireless Assessment of Respiratory and circulatory Distress (WARD) project. This manuscript adheres to the Strengthening the Reporting of Observational Studies in Epidemiology (STROBE) 2007 guidelines.

### 2.1 Participants

The inclusion criteria were age ≥ 60 and scheduled elective major abdominal cancer surgery with an estimated surgical duration of ≥2 h. Exclusion criteria were implanted cardioverter defibrillator or pacemaker, allergy to study devices, severe cognitive impairment assessed by the Mini Mental State Examination (MMSE), or inability to cooperate in wearing the wireless monitoring equipment.^19^

Informed consent was obtained preoperatively, and continuous monitoring was initiated postoperatively after discharge from the PACU and upon arrival at the general ward and continued until discharge or for a maximum of 96 h.

**FIGURE 1:**
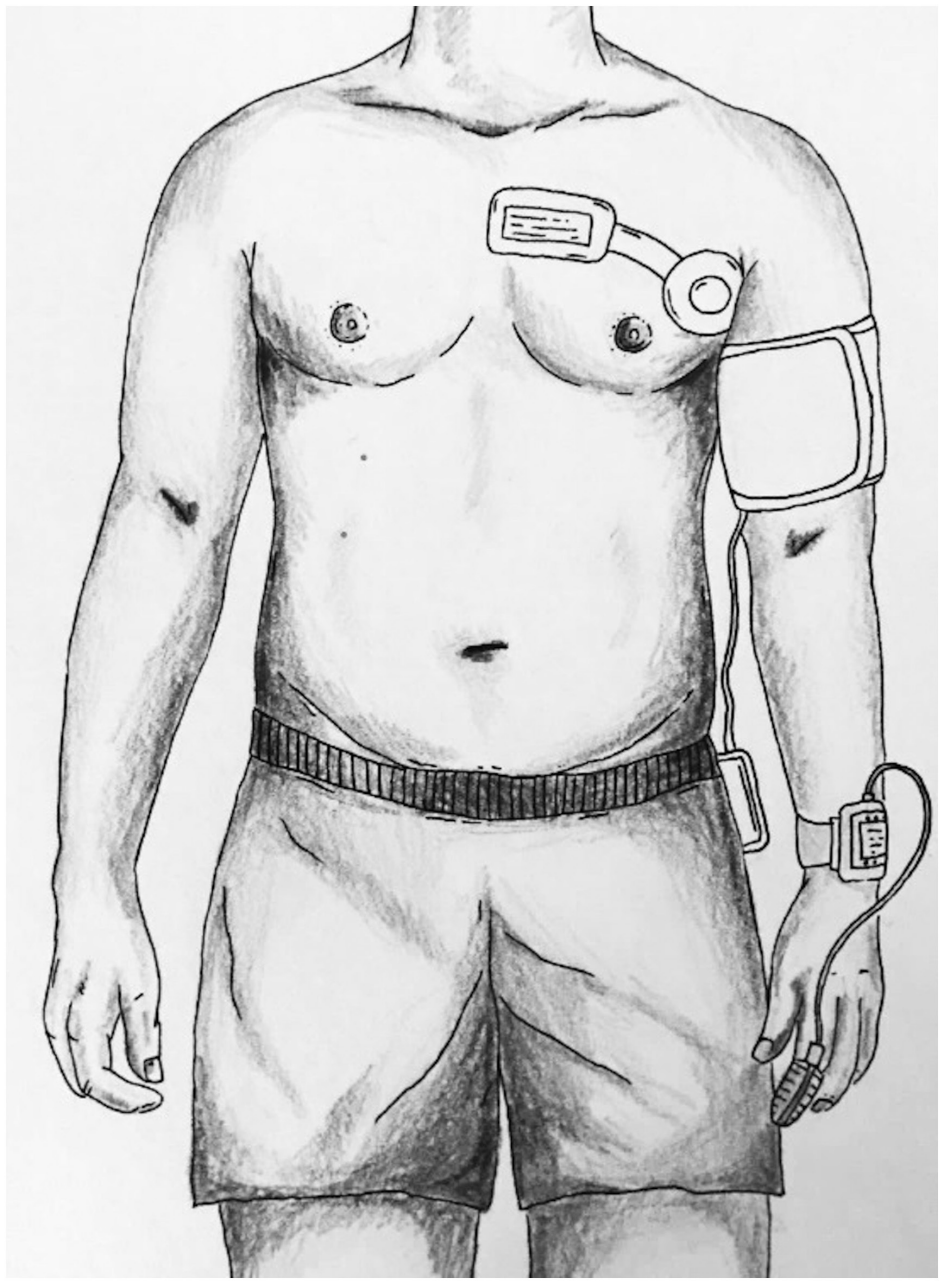
Placement of monitoring devices. Wireless devices to use for monitoring vital signs for up to four days in 491 postoperative patients: A single‐lead‐electrocardiogram, a blood pressure monitor, and a peripheral oxygen saturation. All wireless and transmitting data via Bluetooth to a bedside gateway and onwards to a central server. Illustration courtesy of Liv Brogaard Aasvang

Vital signs were recorded continuously by clinically validated devices^20^; (a) Isansys Lifetouch (Isansys Lifecare, Oxfordshire, UK), an FDA approved electrocardiogram (ECG) patch placed on the thorax with two electrodes (single lead ECG) recording heart rate and respiratory rate continuously; (b) FDA approved Meditech BlueBP‐05 (Meditech Ltd., Hungary), a compact device for non‐invasive oscillometric measurements of blood pressure, in this study programmed to measure every 30 min during the daytime (07 am–9:59 pm) and every 60 min during the nighttime (10 pm–06:59 am) and thus not continuously measured; and (c) FDA approved Nonin WristOx 3150 (Nonin Medical Inc., Minnesota, USA), a wearable finger pulse oximeter for peripheral oxygen saturation (Figure 1). Data were transmitted through Bluetooth to a bedside gateway and from the gateway via a secure hospital wi‐fi connection to a hospital server. Data (except for SpO_2_) were automatically stored in the devices when a patient was out of Bluetooth range from the gateway, enabling the later transfer of data when Bluetooth connection was re‐established.

Clinical ward staff observed and recorded the patients’ postoperative vital signs (EWS monitoring every 8 h or more often) according to usual care. Patients and staff were blinded to values from the continuous monitoring equipment and the perioperative care adhered to the recommendations for enhanced recovery after surgery on each specific ward.^21^ In addition, pre‐operative demographic variables were collected; height, weight, smoking status, alcohol use, American Society of Anesthesiology status (ASA), and pre‐existing medical conditions, including the Charlson Comorbidity Index (CCI).^22^ Vital signs were measured, and the patients performed a Timed Up and Go Test (TUG) to test for basic functional mobility.^23^ The primary outcome was any SAE registered from arrival to the general ward (and wirelessly monitored) until postoperative day 30. A manual for describing and reporting SAEs (and adverse events) was developed for standardization. According to the International Conference on Harmonisation‐Good Clinical Practice (ICH‐GCP‐guideline), SAEs are defined as any untoward medical life‐threatening occurrences, requiring hospitalization or prolongation of existing hospitalization, or results in persistent or significant disability.^24^ In the primary analysis, only the vital signs preceding the first SAE in each patient were included to avoid bias from the impact of the SAE on succeeding vital signs. Furthermore, we recorded serious adverse device effects (SADE), defined as adverse device effects that have resulted in any of the consequences characteristic of a serious adverse event but related to the monitoring devices used in the study.

### 2.2 Variables

Exposure variables were the cumulative duration of vital sign abnormalities and frequency of patients having episodes of predefined vital sign abnormalities. The vital sign thresholds were based on internationally agreed alert thresholds used in track and trigger systems such as the EWS (Figures 3 and 4, Table 2). In addition to the simple 1‐dimensional thresholds, compensation for normal physiological fluctuations over time, and elimination of short durations of minor vital sign abnormalities, we also constructed a 2‐dimensional combination of duration and severity of vital sign abnormalities. For example, to be classified as an episode, SpO_2_ < 92% had to occur constantly for ≥60 min, SpO2 <88% had to happen for ≥10 min, whereas SpO_2_ < 85% had to be present for ≥5 min, and SpO_2_ < 80% was classified as an episode if it lasted longer than one minute. Episodes of vital sign abnormalities occurring less than 5 min apart, were registered as one episode.

Algorithms removed noise and artifacts before analysis. We recorded HR and RR with a one‐minute sampling frequency derived from automatic detection of the QRS complex and R peaks in the single‐lead ECG, digitized at 1000 samples per second. Every minute, 10 s of the ECG was available and assessed if the heart rate was representative or not. The 10‐second segments underwent a computerized filtration process. The signal quality was determined based on correlation analysis between each QRS complex and a template based on the average QRS complex in the segment. At low correlation, the segment would be considered an artifact and thus removed. The HR, RR, and SpO_2_ included both raw values and a calculated average per minute. A SpO_2_ change >4% per second was considered an artifact, and these segments were removed from the final analysis. Blood pressure measurements were assigned for 30 min duration during the day and 60 min during the night. During the computation of episodes of vital sign abnormalities, the last value was carried forward for up to 60 min if data were missing.

### 2.3 Sample size

We estimated that 100 postoperative SAEs would be required to allow future machine learning‐based algorithms. Based on the current literature, describing approximately 20%–30% occurrence of SAEs after major abdominal surgery,^2^, ^3^ we aimed at including 500 patients for analysis. We set a five percent significance level and did no corrections for multiple comparisons due to the exploratory and hypothesis‐generating study design.

### 2.4 Statistical analysis

Patients were divided into three categories: Patients with SAE during monitoring (SAE‐during), patients developing SAE after monitoring (SAE‐after), and patients without SAE (No‐SAE). The relation between the duration and frequency of abnormal vital signs and the primary outcome was analyzed by descriptive statistics, including mean with SD if normally distributed or otherwise as median with IQR. Categorical data were presented as percentages (%). Data were presented per 24 h, but due to the potential impact of circadian rhythm on physiology,^25^ we also stratified data according to day (07.00 am–09.59 pm) and night (10.00 pm–06.59 am). The Kruskal–Wallis test tested the differences in the duration of vital sign abnormalities between the three groups. Frequency of patients with abnormal vital sign episodes (categorical data) was compared between groups by chi‐square test. Due to the difference in monitoring time in patients with SAE during monitoring and those with SAE after monitoring or without SAEs, we normalized data to 24 h to allow comparison between groups:

(1) SAE‐during group: All available data from the 24 h preceding the first SAE were analyzed, and for those patients not having 24 h preceding the SAE, we normalized data to 24 h. (2) For the SAE‐after and No‐SAE groups, data from the entire monitoring period were divided by the monitoring time and multiplied to give the 24‐h average. All analyzes were undertaken using Python v. 3.7.6 (Python Software Foundation) using the packages: pandas v. 1.1.1 (The pandas development team), NumPy v. 1.19.1 (NumPy Developers), and matplotlib v. 3.3.1 (Matplotlib Development Team).

## 3 RESULTS

**FIGURE 2:**
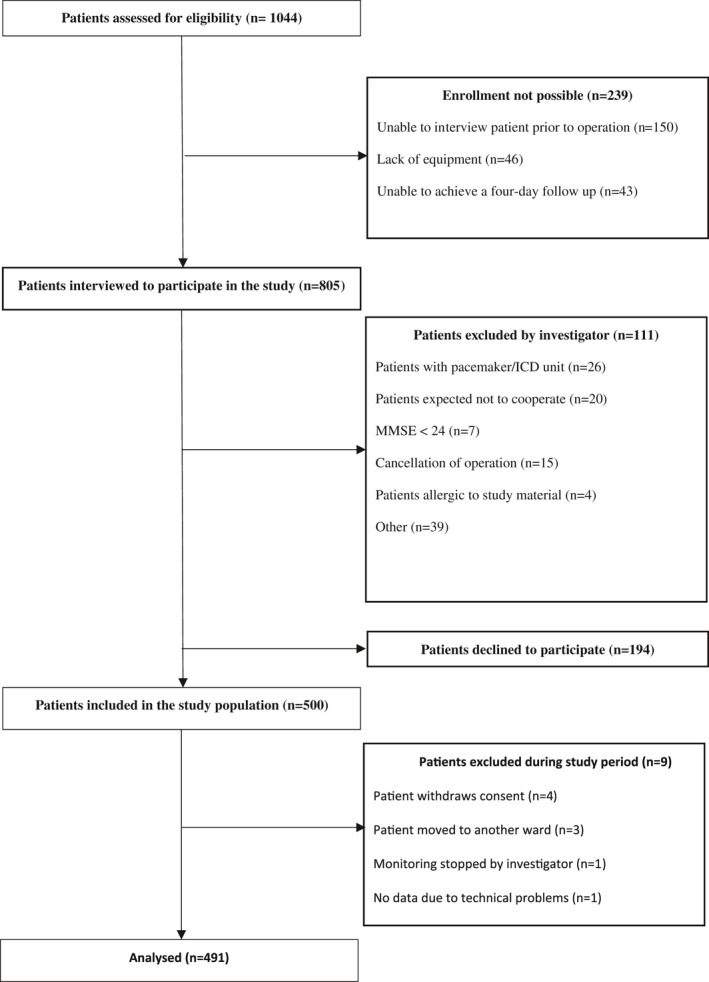
Study flowchart. MMSE: Mini Mental State Examination; ICD: Implantable Cardioverter Defibrillator

Patients were enrolled between February 2018 and July 2020 but halted between March 9, 2020—May 15, 2020, due to the Covid 19 pandemic. Of 1044 patients screened for inclusion, we included 500 patients (Figure 2). A total of nine patients were excluded, resulting in 491 patients in the final analysis. Most patients were men with a median age of 70, a median surgery duration of 3h, 39min, and five patients diagnosed with obstructive sleep apnea (Table 1). The total continuous monitoring time was 34,763 h (median 79h per patient). We recorded a total of 33,464 h of HR and RR data (median 73 h per patient) and 21,531 h of SpO_2_ data (median 43 h per patient) available for analysis. The blood pressure measurements were 33,046 (median 63 times per patient).

**TABLE 1:** Baseline characteristics

Parameter	*n* = 491
Gender, male, female	310 (63%), 181 (37%),
Age, years	70 [66–75]
BMI (*n* = 491)	
<18.5	11 (2.2%)
18.5–24.9	223 (45%)
25–29.9	172 (35%)
≥30	84 (17%)
Smoking history	
Current	63 (13%)
Former	272 (55%)
Never	156 (32%)
Excessive alcohol consumption	96 (20%)
ASA	
I	21 (4.3%)
II	259 (53%)
III	207 (42%)
IV	4 (0.8%)
CCI	
2–3	56 (11%)
4–5	262 (53%)
6–7	126 (26%)
8+	47 (9.6%)
SpO_2_	98% [97–99]
Systolic blood pressure, mmHg	136 [125–149]
Diastolic blood pressure, mmHg	76 [69–84]
TUG (*n* = 453)	
<10 s	396 (87%)
≥10 s	57 (13%)
Hemoglobin, mmol/L (*n* = 461)	7.9 [7.0–8.6]
Creatinine µmol/L(*n* = 466)	77.0 [68–92]
Primary operation:	
Pancreatic resection	166
Bowel resection	152
Esophagus resection	90
Gastrectomy	29
Other major procedures	54
Duration of surgery	3 h 39 min [2 h 32 min–4 h 37 min]
Fluid balance, ml	1240 [862–1503]

Note

BMI: Body Mass Index; kg/(height in m)^2^; Alcohol consumption: Excessive intake is alcohol consumption more than recommended by the Danish Health Authority, which is 24 g/day for men and or 12 g/day for women; ASA: American Society of Anesthesiologist, Physical Status Classification; CCI: Charlson Comorbidity Index; TUG: Timed Up and Go test; SpO_2_: Peripheral oxygen saturation; CI: Confidence interval; Values are given as numbers (percentage) or median of [IQR].

We found that 184 (37%) patients had at least one SAE during the first 30 postoperative days (Table S1). The most frequent first occurring SAEs was surgical site infection (*n *= 45), pleural effusion requiring drainage (*n* = 17), chylothorax (*n* = 16) and bowel obstruction (*n* = 12). The median time from arrival at the general ward to the first SAE was 4 days and 15 h (IQR 2 days, 3 h—8 days, 1 h), and when considering the first SAE, 70 (38%) of these occurred during monitoring. We recorded no SADEs.

### 3.1 Vital sign abnormalities

**FIGURE 3:**
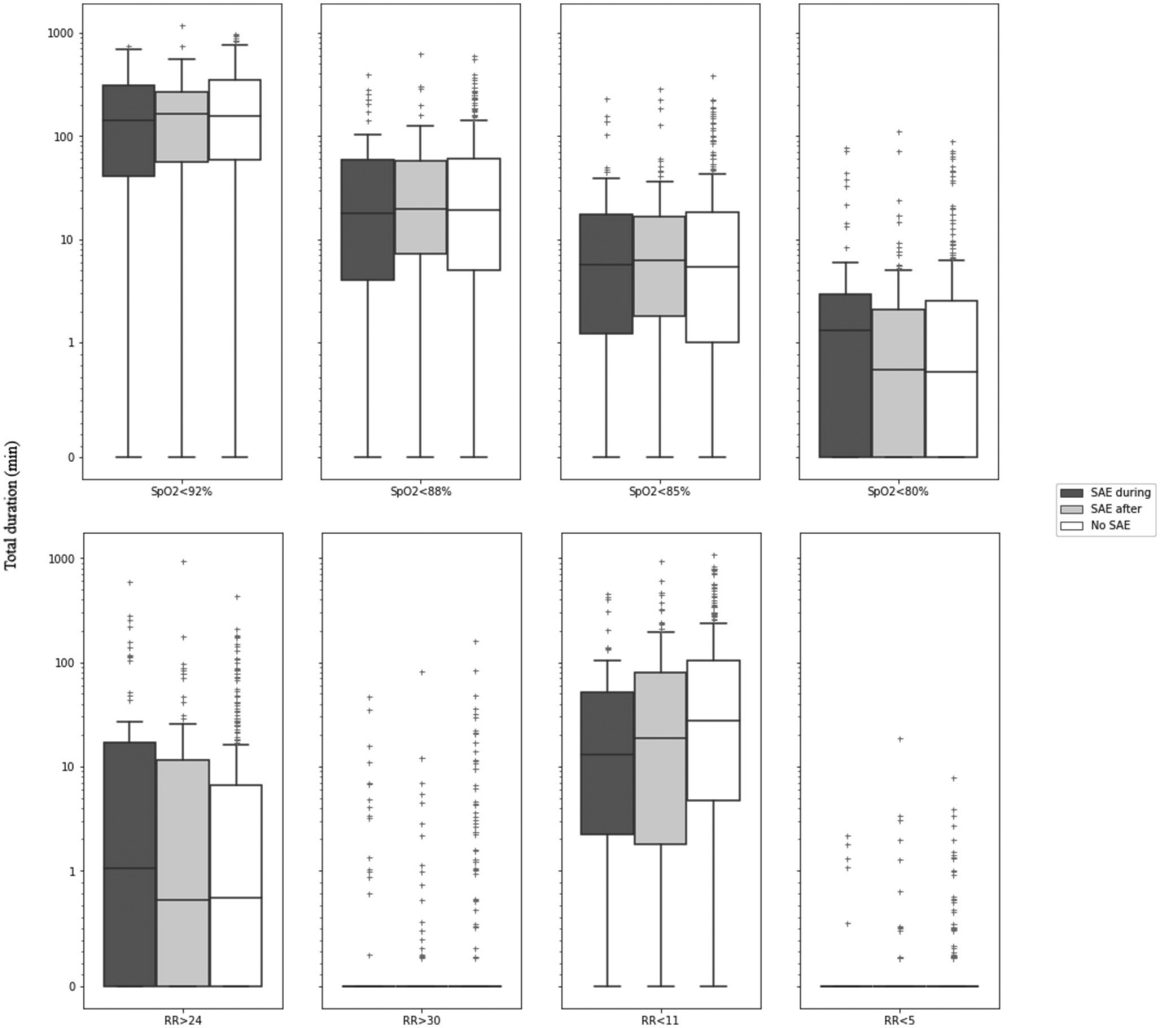
Duration of preceding respiratory vital sign abnormalities in patients with a serious adverse event during monitoring, after monitoring, and patients without any SAE. Boxplots lower box = 25th percentile, median bar = median, upper box = 75th percentile, whiskers 5’th and 95’th percentile, and outliers. SpO_2_: Peripheral oxygen saturation (%). RR: Respiratory rate per min; SAE: Serious adverse event; SAE‐during: Patient group with SAE occurring during monitoring; SAE‐after: Patient group with SAE occurring after monitoring; No‐SAE: Patient group without experiencing any SAE; Y‐axis: Duration (min)

**FIGURE 4:**
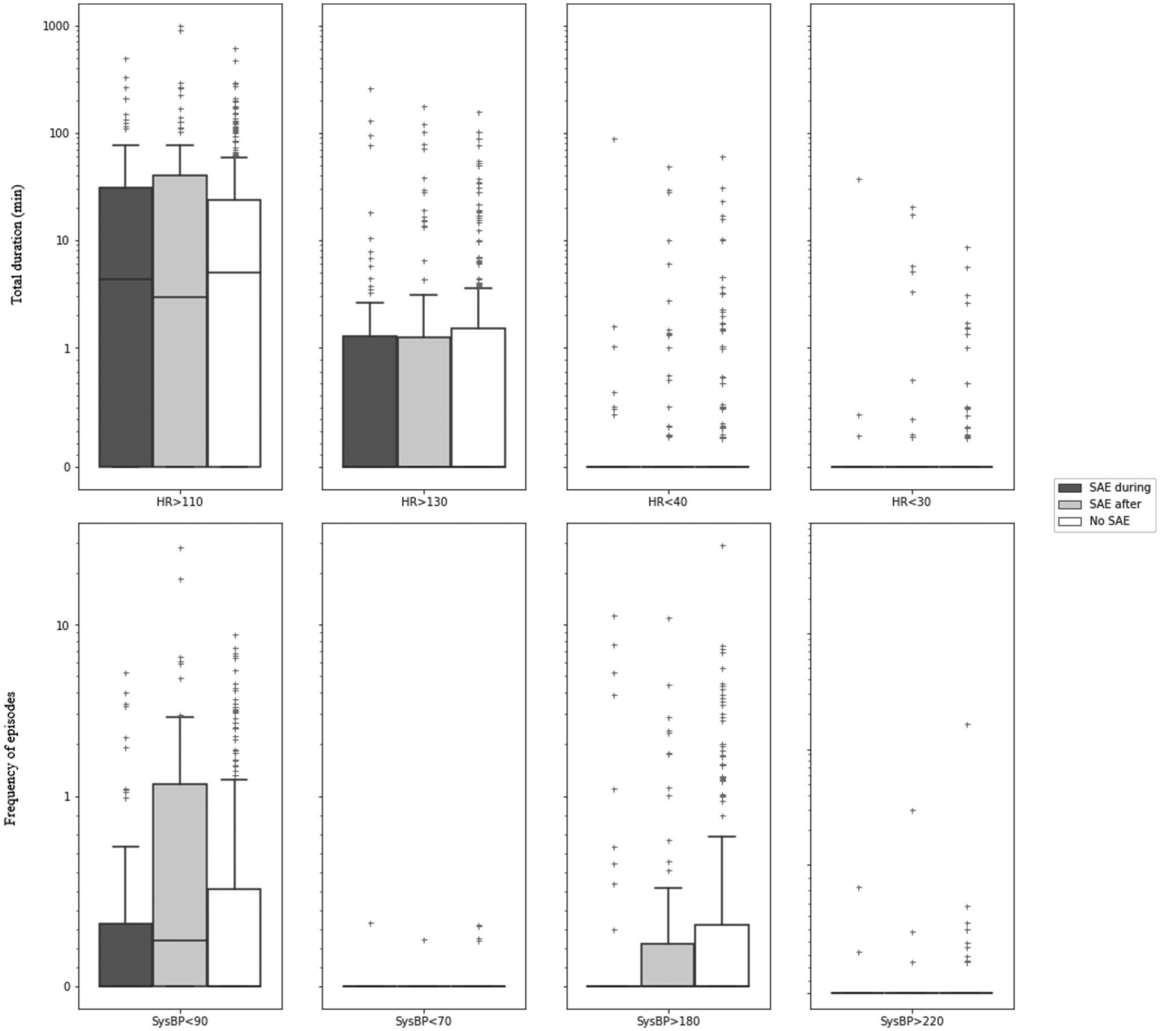
Duration or episodes of preceding circulatory vital sign abnormalities in patients with a serious adverse event during monitoring, after monitoring, and patients without any SAE. Boxplots lower box = 25th percentile, median bar = median, upper box = 75th percentile, whiskers 5’th and 95’th percentile, and outliers. HR: Heart Rate per min; SysBP: Systolic blood pressure (mm Hg); SAE: Serious adverse event; SAE‐during: Patient group with SAE occurring during monitoring; SAE‐after: Patient group with SAE occurring after monitoring; No‐SAE: Patients group without experiencing any SAE; Y‐axis: Duration (min) for HR and frequency of events for SBP

The distribution of time below predefined thresholds is illustrated in Figures 3 and 4 and stratified by day and night in Figures S1 and S2. The frequency of patients experiencing at least one abnormal vital sign episode is presented in Table 2 and Table S2 for day‐night. The duration of vital sign abnormalities occurred for a median of 272 min (IQR 110–447) during the last 24 h in the SAE‐during group compared to 259 min (IQR 153–394) in the SAE‐after group and 261 min (IQR 132 to 468) in the No‐SAE group (*p *= .62).

**TABLE 2:** Frequency of patients with vital sign abnormalities

	Number of patients with episodes of vital sign abnormalities for 24 h (%)		
	SAE during monitoring *n* = 70	SAE after monitoring *n* = 112	No SAE *n* = 309
Respiratory vital sign abnormalities			
SpO_2_ < 92% for ≥60 min	32 (46%)	59 (53%)	160 (52%)
SpO_2_ < 88% for ≥10 min	27 (39%)	60 (54%)	161 (52%)
SpO_2_ < 85% for ≥5 min	25 (36%)	49 (44%)	135 (44%)
SpO_2_ < 80% for ≥1 min	39 (56%)	73 (65%)	206 (67%)
RR < 5 min^−1^ for ≥1 min	4 (5.7%)	5 (4.5%)	8 (2.6%)
RR < 11 min^−1^ for ≥5 min	17 (24%)	36 (32%)	120 (39%)
RR > 24min^−1^ for ≥5 min	15 (21%)	17 (15%)	38 (12%)
RR > 30 min^−1^ for ≥1 min	12 (17%)	8 (7.1%)	32 (10%)
Circulatory vital sign abnormalities			
HR < 30/min for ≥5 min	3 (4.3%)	5 (4.5%)	6 (1.9%)
HR < 40/min for ≥5 min	2 (2.9%)	2 (1.8%)	7 (2.3%)
HR > 110/min for ≥60 min	11 (16%)	8 (7.1%)	12 (3.9%)
HR > 130/min for ≥30 min	5 (7.1%)	4 (3.6%)	12 (3.9%)
SBP < 70 mm Hg ≥two times	1 (1.4%)	0 (0.0%)	0 (0.0%)
SBP < 90 mm Hg ≥ two times	8 (11%)	19 (17%)	29 (9.4%)
SBP > 180 mm Hg ≥ two times	6 (8.6%)	7 (6.2%)	31 (10%)
SBP > 220 mm Hg ≥ two times	0 (0.0%)	1 (0.9%)	1 (0.3%)

Note

Values are numbers (percentage). Data for patients with SAE during monitoring (*n* = 70) were analyzed for vital sign abnormalities in the 24 h preceding the first SAE. Data for patients with first SAE occurring after monitoring (*n* = 112) and patients without SAE (*n* = 309) were analyzed for vital sign abnormalities in 24 h, normalized from the entire monitoring period. Episodes of vital sign abnormalities are normalized to a 24 h period.; a patient with a vital sign abnormality less than one per 24 h will thus not be counted as having a vital sign abnormality. SpO_2_: Peripheral oxygen saturation, RR: Respiratory rate, HR: Heart rate SBP: Systolic blood pressure

#### 3.1.1 SpO_2_

In 470 (96%) patients, SpO2 <92% was registered at least once, with a median duration of 141 min (IQR 41–305), 162 min (IQR 56–269) and 155 min (IQR 59–345) in the SAE‐during, SAE‐after, and No‐SAE groups, respectively, (*p *= .86). Twenty‐five% of patients had cumulative durations of SpO_2_ < 92% lasting longer than 334 min per 24 h. The median duration of desaturation (SpO_2_ < 85%) was 5.7 min (IQR 1.2–17), 6.2 min (IQR 1.8–16) and 5.4 min (IQR 1–18) in the SAE‐during, SAE‐after, and No‐SAE groups, respectively (*p *= .96). Twenty‐five% of all patients had a cumulative duration of SpO_2_ < 85% lasting longer than 18 min per 24 h.

Cumulative duration of SpO_2_ < 80% was longer in patients without SAE’s, but overall, short durations. The medians were 1.3 min (IQR 0.0–2.9), 0.77 min (IQR 0.0–2.1) and 0.74 min (IQR 0.0–2.5) for SAE‐during, SAE‐after, and No‐SAE groups, respectively, (*p *= .54) (Figure 3). There was a high frequency of patients with desaturation episodes, such as SpO2 < 85% for more than 5 min was registered in 25 (36%) SAE‐during, 49 (44%) in SAE‐after, and 135 (44%) in No‐SAE groups, respectively (*p *= .45) (Table 2), with more patients having the episodes of vital sign abnormalities during daytime (Table S2).

### 3.2 Respiration rate

The cumulated median duration of RR < 11 per 24 h was longer in patients without SAEs, (12 min (IQR 2.2–51), 19 min (IQR 1.8–80), and 27 min (IQR 4.7–104) in SAE‐during, SAE‐after, and No‐SAE groups, respectively), however not significant (*p *< .35) (Figure 3). RR <5 or >30 was rare in all three groups without statistically significant differences. The occurrence of RR >24 for more than 5 min occurred in 15 (21%) SAE‐during, 17 (15%) in SAE‐after, and 38 (12%) patients in No‐SAE groups (*p *= .14) (Table 2), with a decrease in the frequency of patients experiencing RR > 24 during the night in all three groups (Table S2).

### 3.3 Heart rate

The cumulative median duration of HR abnormalities was low for all heart rate thresholds. HR > 110 with a median of 4.3 min (IQR 0–31), 2.9 min (IQR 0–40) and 4.9 min (IQR 0–24) in SAE‐during, SAE‐after, and No‐SAE groups, respectively, (*p *= .30), (Figure 4). However, there was a significant difference in the frequency of patients with HR > 110 for more than 60 min, which occurred in 11 (16%) SAE‐during, 8 (7.1%) SAE‐after, and 12 (3.9%) No‐SAE groups, respectively, (*p *< .002). HR < 30, HR < 40, and HR > 130 beats per minute were rare in all three groups (Table 2).

### 3.4 Systolic blood pressure

The number of episodes of hypotension was few in all three patient groups: Systolic blood pressure <90 mm Hg; median 0 episodes/24 h (IQR 0–0.32), 0.24 episodes/24 h (IQR 0–1.2), and 0 episodes/24 h (IQR 0–0.51) in the SAE‐during, SAE‐after and No‐SAE groups, respectively. These results were significantly different (*p *< .01) (Figure 4).

The number of patients with more than two hypotensive events (<90 mm Hg) in a row/24 h was eight (11%) in SAE‐during, 19 (17%) in SAE‐after, and 29 (9.4%) in No‐SAE groups, respectively (*p *= .1) (Table 2). The episodes of blood pressure >180 mm Hg for more than two episodes in a row/24 h was present in six patients (8.6%) in SAE‐during, seven (6.3%) in SAE‐after, and 31 (10%) in No‐SAE groups, respectively (*p* = .48) (Table 2). Patients experiencing systolic blood pressure below 70 mm Hg and above 220 mm Hg were rare in all three groups.

## 4 DISCUSSION

We found no statistically significant association between the total duration of vital sign abnormalities and subsequent occurrence of SAEs. However, episodes of tachycardia were seen more often in patients with SAEs, and the number of hypotension episodes was significantly higher in this group, although rare. The association between hypotension episodes and SAE supports other results linking postoperative hypotension to increased risk of myocardial injury and death.^26^, ^27^

More than one‐third of the patients who underwent major abdominal surgery developed an SAE within 30 days after start monitoring, and episodes of abnormal vital signs were recorded in more than 70% of all patients. Despite only including elective procedures under well‐implemented enhanced recovery set‐ups in the two hospitals, this high frequency of complications following major abdominal surgery confirms findings in previous studies.^2^, ^3^, ^4^ This underlines the need for advancements in perioperative care, including the need for better monitoring, as the current practice of track and trigger systems (EWS) fails to recognize up to 90% of severe abnormal vital signs^16^, ^18^, ^28^, ^29^, ^30^ and thereby maybe failing to improve outcomes.^31^ The detection of abnormal vital signs might benefit from a continuous monitoring system that register, record—and store the measurements allowing looking back in time to evaluate the development of abnormal vital signs or in the future by machine learning assess vital signs longitudinal, thus detecting deteriorating patients.

Apart from failing to recognize critical vital sign abnormalities,^16^, ^18^, ^28^ the manual track and trigger systems are vulnerable to human errors, including adherence to escalation protocols, potentially explaining the lack of impact on morbidity and mortality.^32^ However, a rarely discussed potential explanation may be that the thresholds designed to trigger interventions are not evidence‐based, especially regarding the discrimination towards clinical complications. Our findings suggest that analyzing vital sign data as cumulative durations or frequencies below thresholds is too simplistic. A substantial proportion of patients with severe episodes of abnormal vital signs also have a high frequency of self‐limiting episodes. Thus, we found 17% of patients with severe hypotension in the SAE‐after group versus 9.4% in the No‐SAE group and 21% versus 12% episodes of tachypnea in the SAE‐during versus the No‐SAE group. Although abnormal vital signs may have a considerably different impact on patients depending on their comorbidity status, data suggest that alerts should be context sensitive, for instance, with other alerts depending on activity and time of day.

The findings of a substantial duration of vital sign abnormalities in patients with and without SAEs call for redefining alert thresholds and features with higher sensitivity and specificity for SAEs. This could include the combination of duration and severity, as presented in this manuscript, but with data‐driven iterations of the most predictive combinations and also including trend analyses of the preceding deviating vital signs. Trend analysis may enable a more personalized monitoring system based on individual changes in vital signs over prespecified time courses. Such an alert system will be adaptive to the changes in the patient´s recovery profile and, further, reduce irrelevant alarms.^33^ Based on our findings and similar studies, we believe this is the next logical step in detecting impending complications to allow preventive measures and ultimately develop a relevant clinical alert system. However, before conducting such analysis and subsequent possible implementation of a new monitoring system in the hospitals, we find it essential to assess the clinical effect of such an advanced alert system on patient outcomes.

Establishing evidence for clinically relevant alerts is essential since continuous monitoring will inherently detect a substantial number of abnormal vital signs, supported by our finding, a high occurrence of desaturation episodes despite requesting 60 min of saturation <92%, with no difference in duration or frequency in the three groups. The implications of frequent alerts are of importance. First, there is a risk of alarm fatigue, ignoring even critical alerts.^34^ Second, alerts with low specificity to clinical complications (pneumonia, bleeding, arrhythmias, etc.) may induce unnecessary investigations with the risk of inflicting iatrogenic injury despite self‐limiting and allocating staff to patients without need. In contrast, alarms must not be misinterpreted as insignificant if they represent the first stage of a deteriorating patient, and further research regarding preceding deviating vital signs is needed.

Our study supports that continuous wireless monitoring is feasible, including 92% of the potential observation time with at least one device measuring. However, standard blood pressure monitors were not continuously measuring (measurements every 30 or 60 min), and with the used devices, it would not be possible to measure continuously due to patient discomfort. This requests novel solutions such as algorithms utilizing information from the ECG and plethysmogram to assess blood pressure, or perfusion index, allowing accurate continuous monitoring of the circulation and thereby the opportunity to dismiss the, for some patients, unpleasant standard equipment.^35^, ^36^

The median duration of monitored vital signs differed, with 73 h for HR and RR but only 43 h for SpO_2_. This diversity challenges the integration of measurements. The Nonin device measuring SpO_2_ did not store data when patients were out of range. However, the warmth and discomfort of having a silicon cap attached to the finger for a long time were the main reason for removing the SpO_2_ device. Therefore, our results represent the minimum of abnormal vital signs, and deviations may occur in the periods of missing data, possibly influencing our results. The continued technological development should limit missing data, ensuring better algorithms.

Moreover, future alerts systems should be context‐sensitive, including adapting to the activity and sleep of patients. This is suggested by the finding in our study of differences in day and night, potentially reflecting the circadian rhythm, where identification of patients without this variation could be of particular interest as it may reflect and identify a higher risk of complications.

Although the presented analyses were kept conservative and descriptive, our inclusion of approximately 500 patients and the long monitoring time form a substantial basis for the statistical analyses. We used internationally agreed SAE definitions and a predefined SAE manual to standardize assessments. Thresholds for vital sign abnormalities were based on the EWS, stratifying them into sublevels and assessing the most severe cases by combining time and severity.

The study has limitations. First, we cannot account for medical interventions during monitoring, but the staff was blinded to the continuous data. Second, the time of diagnosis for SAE is might reached several hours after the actual onset, and interventions such as oxygen supplements might have normalized vital signs, hence the 24 h documentation of vital signs before SAE. Some abnormal vital signs might have been missed before SAE, but our standardized outcome assessment was, in our opinion, the best possible methodology. Third, vital sign abnormalities like tachycardia and tachypnea may arise from exercise, but this does not explain the observed periods of desaturation. However, future alarms should ideally compensate for physical activity. Fourth, the included patients underwent major abdominal surgery, and fewerSAEs and vital sign abnormalities are expected in less invasive procedures. Our monitoring period was pragmatically chosen to four days due to the battery time of the ECG patch. However, 75% of SAEs occurred within eight days, and future studies should extend monitoring in the population, even though some studies have shown early deviating vital signs to be associated with long‐term outcomes.^37^ Lastly, we decided to normalize the duration of monitoring to an average of 24 h and frequencies of deviating vital signs to compare the groups, potentially increasing the duration and frequency of abnormal vital signs in the SAE‐after and No‐SAE groups.

In conclusion, the overall duration of abnormal vital signs at prespecified thresholds was not associated with SAE development. There were, however, significantly more more patients with episodes of hypotension and tachycardia before developed an SAE. Thus, future studies should investigate alert criteria with better discrimination for oncoming SAEs.

## CONFLICTS OF INTEREST

CSM, EKA, and HBDS have founded a start‐up company, WARD247 ApS, to pursue the WARD project regulatory and commercial activities. WARD247 ApS has finalized terms for license agreement for any WARD project software and patents. One patent has been filed. CSM reports direct and indirect research funding from Ferring Pharmaceuticals, Merck, Sharp & Dohme Corp., and Boehringer Ingelheim outside the submitted work and lecture fees from Radiometer. EKA reports institutional research funding from Norpharma A/S outside the submitted work and lecture fees from Radiometer. ME has received departmental funding from Merck, Sharp & Dohme Corp outside the submitted work.

## Supporting information

Fig S1

Fig S2

Table S1

Table S2

## References

[1] Joshi GP , Kehlet H . Enhanced recovery pathways: looking into the future. Anesth Analg. 2019;128:5‐7. 30550467 10.1213/ANE.0000000000003746

[2] Pearse RM , Moreno RP , Bauer P , et al. Mortality after surgery in Europe: a 7 day cohort study. Lancet. 2012;380:1059‐1065. 22998715 10.1016/S0140-6736(12)61148-9 PMC3493988

[3] Pearse RM , Clavien PA , Demartines N , et al. Global patient outcomes after elective surgery: prospective cohort study in 27 low‐, middle‐ and high‐income countries. Br J Anaesth. 2016;117:601‐609. 27799174 10.1093/bja/aew316 PMC5091334

[4] Fields AC , Divino CM . Surgical outcomes in patients with chronic obstructive pulmonary disease undergoing abdominal operations: an analysis of 331,425 patients. Surg (United States). 2016;159:1210‐1216. 10.1016/j.surg.2015.11.007 26704782

[5] Weiser TG , Regenbogen SE , Thompson KD , et al. An estimation of the global volume of surgery: a modelling strategy based on available data. Lancet. 2008;372:139‐144. 18582931 10.1016/S0140-6736(08)60878-8

[6] Weiser TG , Haynes AB , Molina G , et al. Size and distribution of the global volume of surgery in 2012. Bull World Health Organ. 2016;94:201‐209F. 26966331 10.2471/BLT.15.159293 PMC4773932

[7] Nepogodiev D , Martin J , Biccard B , et al. Global burden of postoperative death. Lancet. 2019;393:401. 30722955 10.1016/S0140-6736(18)33139-8

[8] Hoogervorst‐Schilp J , Langelaan M , Spreeuwenberg P , et al. Excess length of stay and economic consequences of adverse events in Dutch hospital patients. BMC Health Serv Res. 2015;15:1‐7. 26626729 10.1186/s12913-015-1205-5 PMC4667531

[9] Goldhill DR , White SA , Sumner A . Physiological values and procedures in the 24 h before ICU admission from the ward. Anaesthesia. 1999;54:529. 10403864 10.1046/j.1365-2044.1999.00837.x

[10] Cuthbertson BH , Boroujerdi M , McKie L , et al. Can physiological variables and early warning scoring systems allow early recognition of the deteriorating surgical patient?* Crit Care Med. 2007;35:402‐409. 17205002 10.1097/01.CCM.0000254826.10520.87

[11] Berlot G , Pangher A , Petrucci L , et al. Anticipating events of in‐hospital cardiac arrest. Eur J Emerg Med. 2004;11:24‐28. 15167189 10.1097/00063110-200402000-00005

[12] Smith GB , Prytherch DR , Meredith P , et al. The ability of the National Early Warning Score (NEWS) to discriminate patients at risk of early cardiac arrest, unanticipated intensive care unit admission, and death. Resuscitation. 2013;84:465‐470. 23295778 10.1016/j.resuscitation.2012.12.016

[13] Royal College of Physicians . National Early Warning Score (NEWS) 2: standardising the assessment of acute‐ilness severity in the NHS. Report of a working party | RCP London. R Coll Physicians. 2017; 4‐6.

[14] Pedersen NE , Rasmussen LS , Petersen JA , et al. A critical assessment of early warning score records in 168,000 patients. J Clin Monit Comput. 2018;32:109‐116. 28238106 10.1007/s10877-017-0003-5

[15] Bailey TC , Chen Y , Mao YI , et al. A trial of a real‐time Alert for clinical deterioration in patients hospitalized on general medical wards. J Hosp Med. 2013;8:236‐242. 23440923 10.1002/jhm.2009

[16] Duus CL , Aasvang EK , Olsen RM , et al. Continuous vital sign monitoring after major abdominal surgery‐quantification of micro events. Acta Anaesthesiol Scan. 2018;62(9):1200‐1208. 10.1111/aas.13173 29963706

[17] Verrillo SC , Cvach M , Hudson KW , et al. Using continuous vital sign monitoring to detect early deterioration in adult postoperative inpatients. J Nurs Care Qual. 2019;34:107‐113. 30095509 10.1097/NCQ.0000000000000350

[18] Elvekjaer M , Aasvang EK , Olsen RM , et al. Physiological abnormalities in patients admitted with acute exacerbation of COPD: an observational study with continuous monitoring. J Clin Monit Comput. 2020;34:1051‐1060. 31713013 10.1007/s10877-019-00415-8

[19] Folstein MF , Folstein SE , McHugh PR . “Mini‐mental state”. A practical method for grading the cognitive state of patients for the clinician. J Psychiatr Res. 1975;12:189‐198. 1202204 10.1016/0022-3956(75)90026-6

[20] Elvekjaer M , Carlsson CJ , Rasmussen SM , et al. Agreement between wireless and standard measurements of vital signs in acute exacerbation of chronic obstructive pulmonary disease: a clinical validation study. Physiol Meas. 2021;42:55006. 10.1088/1361-6579/ac010c 33984846

[21] Fearon K , Ljungqvist O , Von Meyenfeldt M , et al. Enhanced recovery after surgery: a consensus review of clinical care for patients undergoing colonic resection. Clin Nutr. 2005;24:466‐477. 15896435 10.1016/j.clnu.2005.02.002

[22] Quan H , Li B , Couris CM , et al. Updating and validating the Charlson comorbidity index and score for risk adjustment in hospital discharge abstracts using data from 6 countries. Am J Epidemiol. 2011;173:676‐682. 21330339 10.1093/aje/kwq433

[23] Richardson S . The timed “up & go”: a test of basic functional mobility for frail elderly persons. J Am Geriatr Soc. 1991;39:142‐148. 1991946 10.1111/j.1532-5415.1991.tb01616.x

[24] European Medicines Agency (EMA) . Guideline for good clinical practice E6(R2). 2018.

[25] Kvaslerud T , Hansen MV , Rosenberg J , et al. Circadian aspects of post‐operative morbidity and mortality. Acta Anaesthesiol Scand. 2010;54:1157‐1163. 20825368 10.1111/j.1399-6576.2010.02296.x

[26] Liem VGB , Hoeks SE , Mol KHJM , et al. Postoperative hypotension after noncardiac surgery and the association with myocardial injury. Anesthesiol. 2020;510‐522. 10.1097/ALN.0000000000003368 32487822

[27] Sessler DI , Meyhoff CS , Zimmerman NM , et al. Period‐dependent associations between hypotension during and for four days after noncardiac surgery and a composite of myocardial infarction and death: a substudy of the POISE‐2 trial. Anesthesiol. 2018;128:317‐327. 10.1097/ALN.0000000000001985 29189290

[28] Sun Z , Sessler DI , Dalton JE , et al. Postoperative hypoxemia is common and persistent: a prospective blinded observational study. Anesth Analg. 2015;121:709‐715. 26287299 10.1213/ANE.0000000000000836 PMC4825673

[29] Taenzer AH , Pyke J , Herrick MD , et al. A comparison of oxygen saturation data in inpatients with low oxygen saturation using automated continuous monitoring and intermittent manual data charting. Anesth Analg. 2014;118:326‐331. 24361847 10.1213/ANE.0000000000000049

[30] Weenk M , Bredie SJ , Koeneman M , et al. Continuous monitoring of vital signs in the general ward using wearable devices: randomized controlled trial. J Med Internet Res. 2020;22:1‐11. 10.2196/15471 PMC7315364 32519972

[31] Petersen JA , Mackel R , Antonsen K , et al. Serious adverse events in a hospital using early warning score – What went wrong? Resuscitation. 2014;85:1699‐1703. 25238741 10.1016/j.resuscitation.2014.08.037

[32] Ronan O’Driscoll B , Bakerly ND , Murphy P , et al. Concerns regarding the design of the bedside monitoring chart for use with the NEWS (National Early Warning System). Clin Med J R Coll Physicians London. 2013;13:319‐320. 10.7861/clinmedicine.13-3-319 PMC5922685 23760715

[33] Brekke IJ , Puntervoll LH , Pedersen PB , et al. The value of vital sign trends in predicting and monitoring clinical deterioration: a systematic review. PLoS One. 2019;14:1‐13. 10.1371/journal.pone.0210875 PMC6333367 30645637

[34] Cropp AJ , Woods LA , Raney D , et al. Name that tone: the proliferation of alarms in the intensive care unit. Chest. 1994;105:1217‐1220. 8162752 10.1378/chest.105.4.1217

[35] Rasmussen PS , Aasvang EK , Olsen RM , et al. Continuous peripheral perfusion index in patients admitted to hospital wards – An observational study. Acta Anaesthesiol Scand. 2021;65:257‐265. 32959371 10.1111/aas.13711

[36] Hill BL , Rakocz N , Rudas Á , et al. Imputation of the continuous arterial line blood pressure waveform from non‐invasive measurements using deep learning. Sci Reports. 2021;11:15755. 10.1038/s41598-021-94913-y PMC8333060 34344934

[37] Bartels K , Kaizer A , Jameson L , et al. HYPOXEMIA within the first three postoperative days is associated with increased one‐year postoperative mortality after adjusting for perioperative opioids and other confounders HHS public access. Anesth Analg. 2020;131:555‐563. 31971921 10.1213/ANE.0000000000004553 PMC7448552

