# Supplementary figures and images for: Continuous monitoring of vital sign abnormalities; association to clinical complications in 500 postoperative patients
Source: Acta Anaesthesiol Scand. 2022 Feb 28;66(5):552–62. doi: 10.1111/aas.14048 (PMC9310747; doi:10.1111/aas.14048)

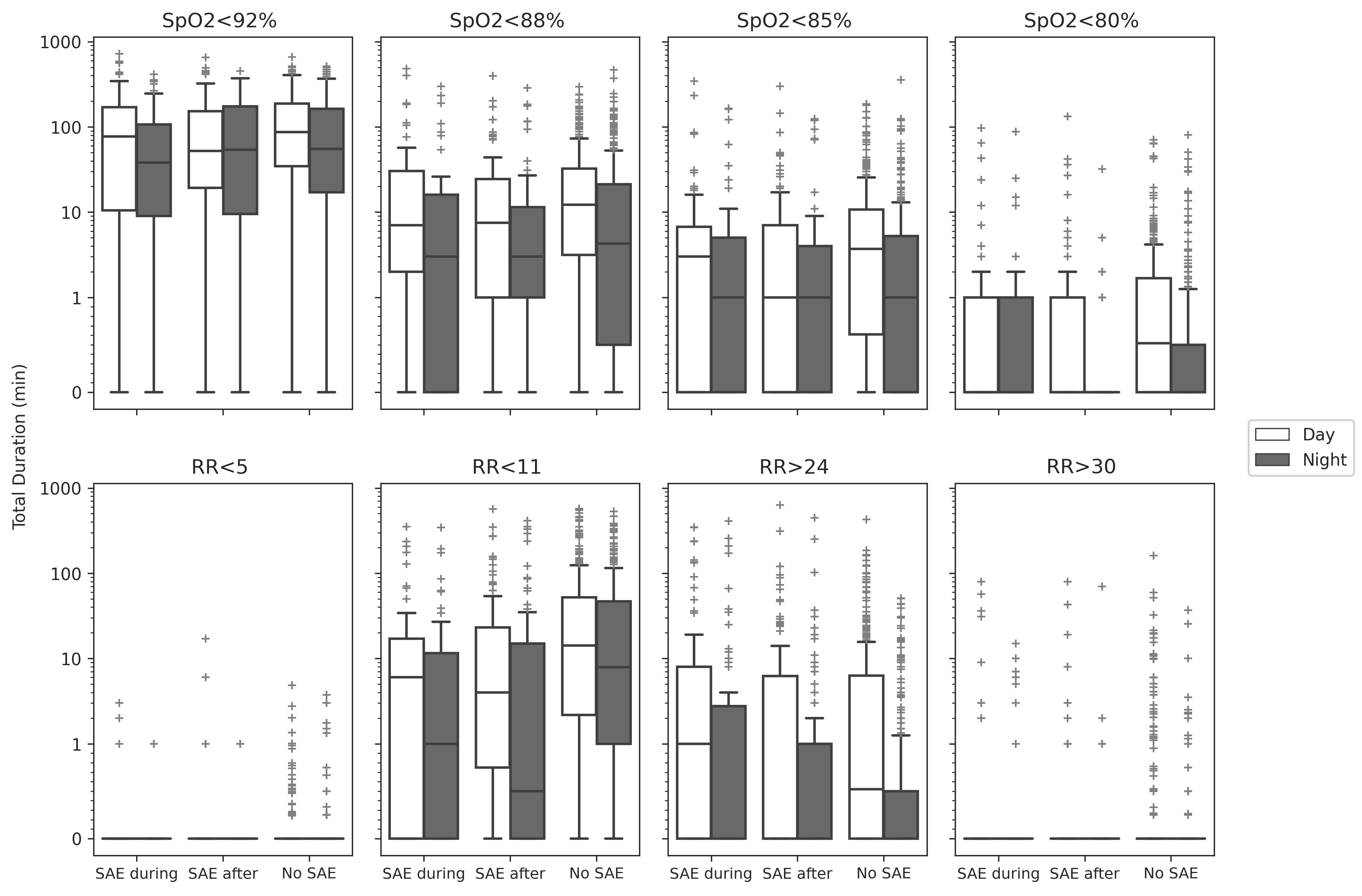

Supplement: Supplementary file 1 — Fig S1 [file AAS-66-552-s004.jpg]

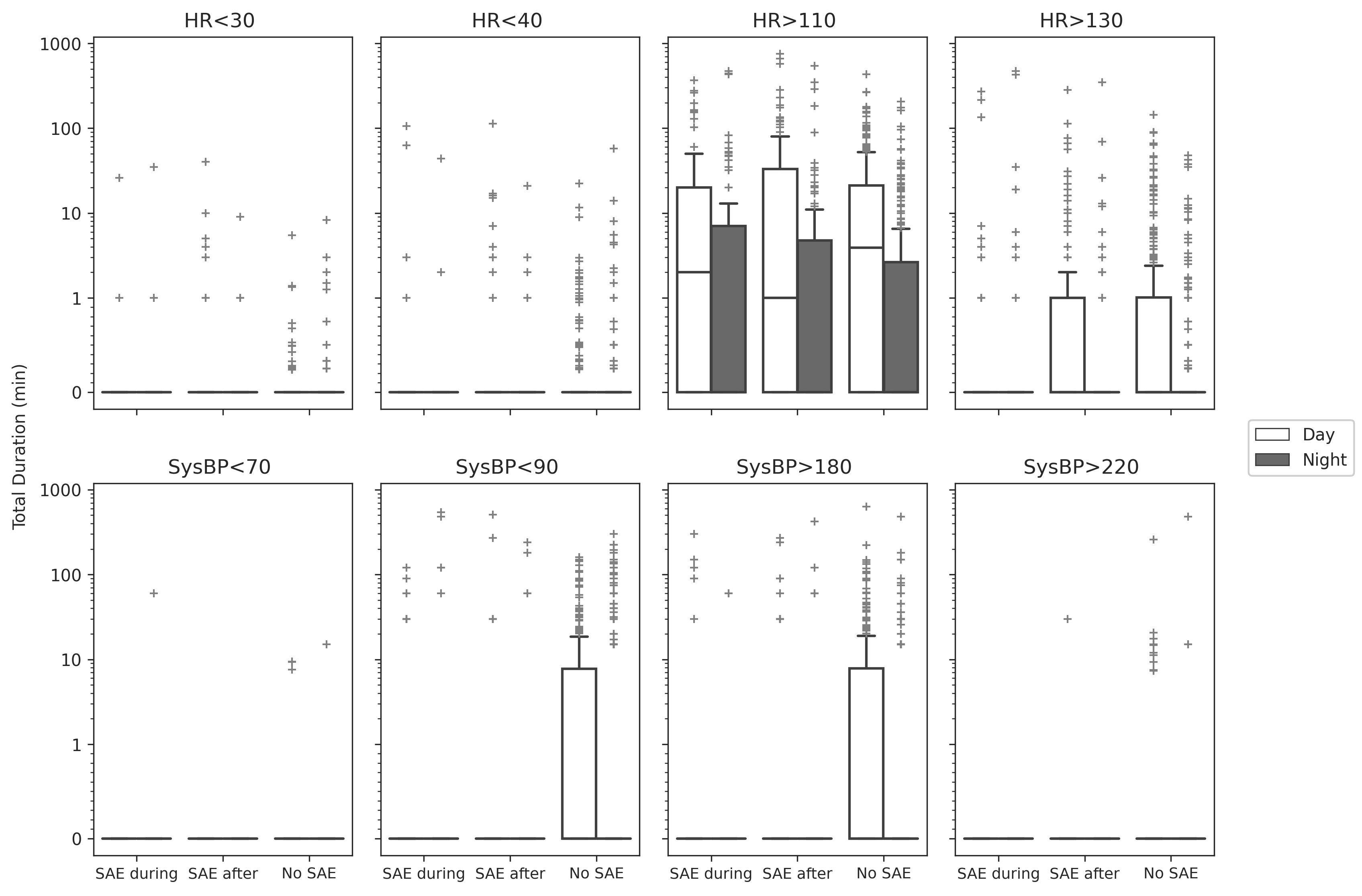

Supplement: Supplementary file 2 — Fig S2 [file AAS-66-552-s002.jpg]
