# Supplementary material for: Continuous monitoring of vital sign abnormalities; association to clinical complications in 500 postoperative patients
Source: Acta Anaesthesiol Scand. 2022 Feb 28;66(5):552–62. doi: 10.1111/aas.14048 (PMC9310747; doi:10.1111/aas.14048)
Supplement: Supplementary file 3 — Table S1 [file AAS-66-552-s003.docx]

Supplemental Table 1. Frequency of patients with serious adverse events categories within 30 days after inclusion.

| **Serious Adverse Events** | **Number of patients (%)**  **(n=491)** |
| --- | --- |
| **Any SAE** | 184 (37%) |
| **All-cause mortality** | 6 (1.2%) |
| **Acute readmission** | 166 (34%) |
| **ICU admission** | 25 (5.1%) |
| **Any neurological SAE** | 9 (1.8%) |
| Delirium | 8 (1.6%) |
| Syncope | 1 (0.2%) |
| Stroke | 0 (0.0%) |
| Transient ischemic attack | 0 (0.0%) |
| **Any respiratory SAE** | 55 (11%) |
| Pleural effusion | 32 (6.5%) |
| Pneumonia | 22 (4.5%) |
| Respiratory failure | 12 (2.4%) |
| Atelectasis | 5 (1.0%) |
| Pneumothorax | 4 (0.8%) |
| **Any cardiovascular SAE** | 40 (8.1%) |
| Heart failure | 14 (2.8%) |
| Atrial fibrillation | 10 (2.0%) |
| Pulmonary embolism | 3 (0.6%) |
| Deep vein thrombosis | 4 (0.8%) |
| Atrial Flutter | 3 (0.6%) |
| Myocardial infarction | 3 (0.6%) |
| Second Degree AV block | 2 (0.4%) |
| Third Degree AV block | 2 (0.4%) |
| Non-fatal cardiac arrest | 1 (0.2%) |
| Other supraventricular tachycardia | 1 (0.2%) |
| Ventricular tachycardia | 1 (0.2%) |
| **Any infectious SAE** | 87 (18%) |
| Surgical Site Infection | 73 (15%) |
| Sepsis | 24 (4.9%) |
| Septic shock | 9 (1.8%) |
| Urinary tract infection | 7 (1.4%) |
| **Any other SAE** | 93 (19%) |
| Other SAE | 65 (13%) |
| Bowel Obstruction | 24 (4.9%) |
| Acute renal failure | 5 (1.0%) |
| Hypoglycemia | 1 (0.2%) |
| Diabetic Ketoacidosis | 1 (0.2%) |
| Fracture | 1 (0.2%) |
| Opioid intoxication | 1 (0.2%) |
| Major bleeding | 9 (1.8%) |
| **Interventions** | 110 (22%) |
| Reoperation | 67 (14%) |
| Surgical drainage | 66 (13%) |

Values are numbers (percentages). The diagnosis had to be a new-onset or apparent worsening of symptoms. If the criteria for a specific SAE were met before inclusion, it was adjudicated as present at baseline and not included in the analysis. Some patients had more than one SAE included in the analysis. SAE; Serious Adverse Event.
