# Supplementary material for: Continuous monitoring of vital sign abnormalities; association to clinical complications in 500 postoperative patients
Source: Acta Anaesthesiol Scand. 2022 Feb 28;66(5):552–62. doi: 10.1111/aas.14048 (PMC9310747; doi:10.1111/aas.14048)
Supplement: Supplementary file 4 — Table S2 [file AAS-66-552-s001.docx]

Supplemental Table 2. Frequency of patients with episodes of vital sign abnormalities, stratified by day- and night.

|  | Number of patients with episodes of vital sign abnormalities for 24 hours (%) | | | | | |
| --- | --- | --- | --- | --- | --- | --- |
|  | SAE during monitoring  n=70 | | SAE after monitoring n=112 | | No SAE n=309 | |
|  | Day | Night | Day | Night | Day | Night |
| Respiratory vital sign abnormalities |  |  |  |  |  |  |
| SpO2 < 92 % for ≥ 60 min. | 26 (37%) | 19 (27%) | 44 (39%) | 13 (12%) | 100 (32%) | 66 (21%) |
| SpO2 < 88 % for ≥ 10 min. | 24 (34%) | 15 (21%) | 49 (44%) | 23 (21%) | 118 (38%) | 64 (21%) |
| SpO2 < 85 % for ≥ 5 min. | 20 (29%) | 14 (20%) | 41(37%) | 11 (9.8%) | 100 (32%) | 42 (14%) |
| SpO_2_ < 80 % for ≥ 1 min. | 37 (53%) | 19 (27%) | 63 (56%) | 22 (20%) | 176 (57%) | 74 (24%) |
| RR < 5min^-1^ for ≥ 1 min. | 2 (2.9%) | 2 (2.9%) | 4 (3.6%) | 0 (0.0%) | 4 (1.3%) | 4 (1.3%) |
| RR < 11 min^-1^ for ≥ 5 min. | 12 (17%) | 10 (14%) | 27 (24%) | 26 (23%) | 89 (29%) | 84 (27%) |
| RR > 24 min^-1^ for ≥ 5 min. | 13 (19%) | 9 (13%) | 12 (11%) | 8 (7.1%) | 34 (11%) | 11 (3.6%) |
| RR > 30 min^-1^ for ≥ 1 min. | 9 (13%) | 9 (13%) | 7 (6.2%) | 3 (2.7%) | 24 (7.8%) | 7 (2.3%) |
| Circulatory vital sign abnormalities |  |  |  |  |  |  |
| Heart rate < 30 min^-1^ for ≥ 5 min. | 2 (2.9%) | 2 (2.9%) | 5 (4.5%) | 1 (0.9%) | 2 (0.6%) | 3 (1.0%) |
| Heart rate < 40min^-1^ for ≥ 5 min. | 1 (1.4%) | 2 (2.9%) | 2 (1.8%) | 0 (0.0%) | 4 (1.3%) | 2 (0.6%) |
| Heart rate >110 min^-1^ for ≥ 60 min. | 7 (10%) | 5 (7.1%) | 5 (4.5%) | 2 (1.8%) | 9 (2.9%) | 1 (0.3%) |
| Heart rate >130 min^-1^ for ≥ 30 min. | 4 (5.7%) | 3 (4.3%) | 3 (2.7%) | 0 (0.0%) | 9 (2.9%) | 0 (0.0%) |
| SBP < 70 mmHg ≥ One time | 0 (0.0%) | 1 (1.4%) | 0 (0.0%) | 0 (0.0%) | 0 (0.0%) | 0 (0.0%) |
| SBP < 90 mmHg ≥ One time | 8 (11%) | 4 (5.7%) | 7 (6.2%) | 4 (3.6%) | 17 (5.5%) | 13 (4.2%) |
| SBP > 180 mmHg ≥ One time | 5 (7.1%) | 2 (2.9%) | 6 (5.4%) | 0 (0.0%) | 20 (6.5%) | 2 (0.6%) |
| SBP > 220 mmHg ≥ One time | 0 (0.0%) | 0 (0.0%) | 0 (0.0%) | 0 (0.0%) | 1 (0.3%) | 0 (0.0%) |

Values are numbers (percentage). The abnormality had to be measured contiguous for the predefined time to count as a vital sign abnormality. Data for patients with SAE during monitoring (n=70) were analyzed for vital sign abnormalities in the 24 hours preceding the first SAE. Data for patients with first SAE occurring after monitoring (n=112) and patients without SAE (n=309) were analyzed for vital sign abnormalities in 24 hours, normalised from the entire monitoring period. Vital signs abnormalities during daytime were analyzed for 899 min. (07:00 am-09:59 pm) and vital sign abnormalities during the night-time (10:00 pm-06:59 am) was analyzed for 539 min. Episodes of vital sign abnormalities are normalized to a 24 h period and stratified at day-night.; a patient with vital sign abnormality less than one at day – or night will thus not be counted as having a vital sign abnormality.
